# Alveolar Abscess

**Published:** 1882-04

**Authors:** A. G. Friedrichs


### ARTICLE II.

### *Alveolar Abscess.*

BY A. G. FRIEDRICHS, M. D.

The limited time accorded me for preparation prevents the bestowal upon the subject in hand that attention and elaboration which it deserves. However, it shall be my endeavor to present it in such a manner as to elicit an interest in a malady so prevalent and yet so generally little understood.

Alveolar abscess is a collection of pus in a sac, found in the socket of a tooth at the extremity of the root. The time required for its formation ranges from three to fifteen days. Its history generally begins with a feeling of fullness and tension, succeeded by a dull, heavy, annoying pain in the parts affected. As the disease progresses, the pain increases; the tooth is loosened and elevated and so sore that the slightest touch is unbearable—in fact the whole attention of the patient seems to be directed to the avoidance of contact. The inflammation, first affecting the periosteum of the tooth, extends to the gums and surrounding parts. The gums around the affected tooth become dark red, swollen and painful. Considerable constitutional disturbance is exhibited in some cases. Elevation of temperature with a hot and dry skin, with a bounding pulse; breath offensive and tongue thickly coated; in other words an inflammatory fever. With the increasing inflammation of the periosteum of the root, an effusion of coagulable lymph takes place, which hardening attaches itself to the root around the apex and ultimately a sac is formed. Then suppuration takes place, and if there is no exit for the pus, either through the root canal and pulp cavity or along the side of the root to the surface of the gums, one side of the alveolus, especially in debilitated persons, undergoes a complete resorption from the proliferation of the connective tissue cells, and an exit is thus formed through the socket and gum for the escape of the pus. A direct lateral passage is not always effected through the gum and alveolus. It is not an infrequent occurrence for a purulent infiltration into the spongy osseous tissue surrounding the alveoli to take place, and the more aboundant the tissue, the greater the liability to the occurrence of infiltration. The maxiliary periosteum being once perforated the necrotic pus flows downward by its own gravity into the loose connective tissue, unless its course is obstructed, when it seeks an outlet in another direction, which is guided by the local anatomical relations. The fistulous track opening either in the mouth or upon the cheek, as it not unfrequently happens, attains a considerable length, terminating in a fistulous ulcer upon the external integument.

This disease allowed to continue and not arrested will continue for years, producing very serious complications and even jeopardizing life. Physicians do not generally recognize its pernicious effects and treat that with levity, which demands a thorough investigation of all the parts involved in the region of the oral cavity, and which operative inferences alone can remedy.

The causes for the production of alveolar abscess may be stated as: Caries, which occasions the inflammation of the pulp, and through it by direct transmission of the irritation from the inflamed vessels and nerves to those of the periosteum of the root; the inhalation of the phosphorous fumes; abuse of mercury; dead and dying pulps confined in the pulp chamber; imperfect filling; filling upon exposed pulp; ligatures around the necks of the teeth; foreign bodies may be forced into the pouches formed by the gums, viz.: bits of tooth-picks; stumps of teeth; concussion of the jaw from a fall, blow or kick; improper occlusion where the pressure in mastication is exerted unevenly, and in fact and condition that could produce an inflammation of the root membrane. The rheumatic, gouty and scrofulous diatheses is also a predisposing cause.

Abscesses occurring upon the root of the upper incisors usually perforate the anterior wall of the alveoli and the fistulous tract opens upon the labial side of the gums, a little below the focus of suppuration. Sometimes the abscess takes an upward course and opens into the usual cavity. Not unfrequently the pus sac extends downwards and backwards perforating the hard palate, leaving a round hole of considerable size. It also happens that the same abscess will spread out in all three directions perforating the usual labial and palatal surfaces.

Dr. Bond describes a case of alveolar abscess which came under his observation thus:

"The patient was a lady about 30 years of age. She had been suffering for some twelve months with a continual discharge of pus from the velum palati. Becoming alarmed at the continuance of the discharge, she called in her family physician, who, after examining it closely to ascertain the place whence the discharge came, soon satisfied himself that it was from the socket of a diseased tooth. Passing his hand along the superior alveolar border, he discovered a protrubrance about the size of a hazel-nut upon the back of each of the central incisor teeth. The extraction of the four named teeth was advised, and when removed was found to be considerably necrosed. The discharge immediately stopped. The pus, instead of passing out through the nasal plates of the superior maxilla, passed back over the roof of the mouth and escaped as described."

My father has now a patient under treatment where the whole anterior wall of the alveoli of an upper lateral incisor and gum have sloughed away, leaving a fistulous opening into which a pea could be easily inserted.

The upper canines are less frequently the seat of disease. When they become affected, it is generally an extension from the lateral incisors or bicuspids.

Prof. Sewell reports a case of fistulous opening at the inner canthus of the right eye of a child 10 years of age. There had been a constant purulent discharge from the opening for some months, and to all appearances resembled a lachrymal fistula. The sound was passed into the opening and it extends as far as the canine tooth, which was discolored. It was extracted, and recovery speedily ensued.

Mr. Fleshman relates a case of a fistulæ opening in the nasal cavity. A little girl of five years was troubled with a constant though not profuse discharge of semi-purulent mucous from the right nostril and appeared to be a sequel of a cold. The mucous membrane was healthy, and there were no indications of a morbid growth. "Having baffled all kinds of treatment, I suspected," says Mr. F., "there must be some undiscovered local irritation." Not being able to find anything wrong in the nasal passages, he looked to the condition of the teeth. Finding a right upper canine carious, extracted it. The discharge lessened on the following day and in a day or two disappeared altogether.

When the upper bicuspids and molars become affected the fistulæ, as a general rule, open anteriorly; but they likewise perforate the alveolar process, and once the bone is undermined the pus forces its way along the connective tissue sheaths of the muscles, and escapes externally through the integument, or penetrates the parotid gland and produce a salivary fistulæ. Should the roots extend into the antrum the pus will discharge into that cavity, and when the teeth or roots are extracted they will leave a fistulæ of the antrum.

Mr. J. A. Salter relates the following grossly neglected case of inflammation of the root membrane of an upper molar. A female, aged twenty-four, was attacked with a severe toothache, referred to the first upper molar on the right side—the pain being accompanied by an extensive swelling of the face and attended with intense suffering. The eye-ball became protruded, and she soon noticed she was unable to see with that eye. In a short time after this the abscess pointed in the vicinity of the inner, and later near the outer canthus, and a large quantity of pus escaped. The openings then closed again, and the general symptoms remained the same. The latter condition continued for three weeks. On admission to the hospital, the patient presented a repulsive disfigurement of the face, with œdema of the lids, and livid skin. The first molar on the right side, together with other carious teeth, were removed, and the antrum could be reached through the partially absorbed alveoli of the first-named tooth. There was considerable necrossed bone, including a large portion of the inner and outer wall of the orbit which was separated. Mobility of the iris was restored, but not vision.

Pollock relates a similar case in his practice, where there was an extensive inflammation of the whole maxillary region, involving also the organs of the orbit. The inflammation subsided, but sight was lost.

Mr. Smith, in a lecture on Alveolar Abscesses, gives the following case: A few years ago, a middle-aged man asked him his opinion about a sore on his cheek, midway between his mouth and ear. A probe was introduced, and it was found that it came in contact with the fangs of the last upper tooth, and in ten days after its extraction he was entirely relieved.

C. Williams speaks of a case of alveolar abscess which occurred subsequent to the extraction of an upper molar and opened upon the inferior margin of the orbit just beneath the outer commisure of the eyelid. Pus made its way beneath the zygomatic process, along the temporal muscles and its escape being prevented by the temporal fascia continued through the spheno maxillary fissure into the outer and lower portion of the orbit. There was marked opthalmia of the left eye combined with serious chemosis. The pus was evacuated by means of incisions in the temporal region. An improvement followed immediately.

Garretson states that in consultation with one of his confreres he saw a young lady of nineteen summers who had a fistulæ right in the centre of the hard palate which had annoyed her for two years. On examining the mouth the dentures were so complete as to have escaped notice—all the teeth being perfect excepting a single molar, which tooth had a small filling of gold on its grinding surface. Six days after extraction the patient was dismissed cured.

The fistulous tracks due to suppurative inflammation of the periosteum of the roots of the lower teeth have no favored or special location to make their exits. They may open upon the chin, neck, in front of or behind, or in the ear itself, in the cervical region, upon the nape of the neck or the thorax. Sometimes the formation of these abcesses are productive of very injurious and alarming consequences, even at times resulting in death. I will relate a few cases in illustration.

A case was sent to me from Touro Hospital by Dr. Loeber. The patient had suffered for 18 months, and from his own statement was thereby incapacitated from earning his livelihood for the previous six months. He had been in the hospital in Cincinnati, O., had been examined by some of the faculty of the Cincinnati Medical College, but the extraction of the offending tooth was never suggested until Dr. Loeber saw him. The abscess had refilled several times, had been opened and had opened itself in the angle of the jaw, but these had closed and the fistulous track had extended from the offending organ (which was an impacted lower wisdom tooth) to the first molar, where it was continually discharging. I extracted the tooth and afterward removed a sequestrum fully a half inch long. I saw him several days afterwards when he appeared to be doing well. I have not seen him since.

Prof. Strasky, in communication to Prof. Wedl, writes the following:

"Several years ago I was consulted by an elderly lady in regard to a set of false teeth. Presently, to my surprise, she began to arrange the dressing of a purulent ulcer upon the left side of the chest, opposite the armpit, in the region of the 4th or 5th rib. To my question as to what ailed her, she replied that she had been under care of the most noted physicians in the city, who had treated this ulcer with all sorts of salves and plasters, but to no purpose, for the ulcer began as a small abscess, gradually increasing in size and became more painful. When I examined the mouth for the purpose of fitting the set of teeth, I found the left lower wisdom tooth deeply imbedded within the gums surrounding it; the crown was quite destroyed by caries; the gums around it were detached, sensitive, and upon pressure, pus oozed out from them. As I had previously conjectured that the ulcer upon the thorax was dependent upon an affection of the tooth,—I applied pressure from the angle of the jaw along the surface of the neck to the region of the ulcer, and became satisfied that pus escaped from two points of the ulcerated surface. The carious tooth was extracted and the ulcer healed in the course of a few weeks."

Dr. Harris relates the following case: A middle-aged lady had been suffering for four years with a fistulæ at the apex of the chin. During this period she had consulted some five or six different physicians, and had been operated upon twice for a supposed disease of the bone. Examining the mouth there was not a carious tooth, but upon striking them separately with a steel instrument, the left lateral lower incisor was painful.

Pagello had a case under treatment where the fistulæ had likewise opened upon the dimple of the chin. He injected an infusion of madder in the fistulous canal, and in a few days the incision above it became discolored. The offending member being removed, it speedily healed.

Dr. Bell relates a case where he was sent for in great haste to see a physician—a Dr. E., who resided some distance in the country. The Doctor was taken some two weeks previous with a tooth-ache in the left lower *dens sapientiæ.* A neighboring physician was called in; but he had diagnosed it neuralgia and made no effort to extract the tooth. The inflammation now extended rapidly to the fauces, tonsils and muscles of the neck, jaw and face. Obstructed deglutition and a high fever soon supervened. Blood letting, cathartics and fomentatives to the face gave no relief. Respiration was difficult, and the muscles of the jaw became so rigid and contracted that his mouth could not be opened. Such was the condition of the patient when Dr. B. saw him. The fever was then succeeded by alternate paroxysms of cold and heat. Efforts to sufficiently open his mouth by forcible means proved ineffectual. While his jaws were thus partially separated, he attempted to swallow some warm tea. In the effort the abscess bursted and discharged nearly a whole table-spoonful of pus through his mouth, and it is supposed that nearly double that quantity went down into his stomach. This gave him immediate relief, but it was not until the next evening that the jaw could be forced open sufficiently to permit the extraction of the tooth. To the roots of this tooth, which were united, was a sac about the size of a large pea, filled with pus. Two weeks afterwards he had entirely recovered.

I, myself, came across a similar case but with this addition, the sac had also opened upon the cheek.

Lynseele described a case of meningo-encephalitis which resulted in death, caused by an abscess which formed upon the roots of a lower molar wisdom tooth, the crown of which had been broken off in an ineffectual attempt at extraction. The pus worked its way along the bone (which became in consequence denuded) upon the inner surface of the ramus of the jaw to the base of the cranium; it then entered the cranial cavity through the foramen ovale spinosum and rotundum, where it spread out upon the base of the brain, becoming the origin of the meningo-encephalitis.

A very curious result of alveolar abscess is the formation of osseous cysts on the sides of the jaw; the pus, instead of inducing absorption, is provided for by the expansion of the outer plate of the bone. There is a little or no sense of fluctuation or crackling on pressure; no appearance of inflammation in the surrounding soft parts. They usually form very rapidly—reach the size of a hickory nut in a few months. This rapid growth is especially diagnostic. Extraction of the tooth is the simplest remedy, but a hole might be bored through the cyst and treated by injections of iodine.

Abscesses associated with the temporary teeth are of special importance. The excitability and irritability of the young jaw, besides inflammatory affections generally run a more rapid course with children than with older people, particularly in a case in which development takes place within small limits, with comparative rapidity, may prove more than the parts can antagonize, resulting in an extensive disorganization, and the more extensive the infiltration the greater the danger that a large portion of the jaw will become necrosed.

Children of a scrofulous or tuberculous diathesis are more liable to the occurrence of partial necrosis of the jaw, from the fact that in them the infiltration undergoes a speedy degeneration on account of the rapid proliferation of the elementary organs.

During the shedding of the temporary teeth, when children are attacked with any of the inflammatory fevers and are at the same time subject to the above mentioned diathesis, then are they most liable to periostitis and necrosis of the jaw.

Salter, in an article on "Surgical Diseases connected with the Teeth," published in the 4th volume of Holmes' Surgery, speaks of two cases of necrosis of the jaw after variola; five after measles: fifteen after scarlet fever; and most of these cases occurred in children five years of age.

Should abscesses in the temporary teeth be associated with any of the exanthems, they should be immediately removed. Infantile abscess should never be allowed to run on; should they not be immediately responsive to modification, remove the offending tooth without delay.

We should also be on our guard and not make a too hasty diagnosis, as there have been several cases of aneurism of the superior palatine artery.

Tertiary syphilis always produces an inflammation of the palatal periosteum, and if not combatted remedially, results in the loss of the palatal bones.

As regards the treatment of alveolar abscess, it should be preventative rather than curative, as it is seldom the case that the integrity of the parts are so well restored as to prevent a recurrence of the disease. The opening in the gum may close and the pus may cease secreting, but any derangement of the general system may induce an irritation of the alveolar dental periosteum and occasion a recurrence of the disease. Prompt antiphlogistic treatment should be immediately resorted to; applications of leeches to the gums, scarification of the surrounding parts and systemic depletion by means of saline cathartics. Should these fail, remove the tooth. Should the tooth on the other hand, on account of its location or for other reasons, be valuable, and the patient be willing to submit, warm fomentations should be applied to the gums; never, however, should these fomentations and emolient poultices be applied externally, as they are rarely productive of any advantage and may do harm by promoting the discharge of matter through the cheek or lower part of the face. When this does occur, after the orifice is closed there remains a puckering of the skin which quite disfigures it. By warming a preserved fig and placing it upon the gum of the tooth affected will answer the purpose; excise the gum and bore through the alveolar wall to the seat of the disease, thus making an exit for the pus. In all cases the indications both local and general, are to be appreciated and recognized. Remove whatever may be the cause—if it be a dead pulp, extract it; if it be pent-up gas in the pulp-chamber, bore a hole into it, and so on, and when every other expedient is exhausted then remove the tooth. Abscesses affecting the lower wisdom teeth, should they be at all persistent, the wisest and safest treatment is extraction.

In reviewing the cases above cited, the very evident fact reveals itself, that in every instance, where serious complications have arisen, they were invariably due to a failure to recognize correctly the cause of the disorder. The plea that they did not fall into reliable and otherwise enlightened hands, the report fully contradicts, and it is a melancholy reflection that the medical fraternity, in its eagerness to investigate and combat the greater evils, ignore the claims of the lesser ones, apparently forgetting that in them often are implanted the germs of disease which flourish, undermining the vital fabric, while they seek for other causes until death—a merciful visitor to the sufferer—relieves them of all future care.—*Herald of Dentistry.*

